# Cellular growth defects triggered by an overload of protein localization processes

**DOI:** 10.1038/srep31774

**Published:** 2016-08-19

**Authors:** Reiko Kintaka, Koji Makanae, Hisao Moriya

**Affiliations:** 1 Graduate School of Natural Science and Technology, Okayama University, Okayama, Japan; 2 Research Core for Interdisciplinary Sciences, Okayama University, Okayama, Japan.

## Abstract

High-level expression of a protein localized to an intracellular compartment is expected to cause cellular defects because it overloads localization processes. However, overloads of localization processes have never been studied systematically. Here, we show that the expression levels of green fluorescent proteins (GFPs) with localization signals were limited to the same degree as a toxic misfolded GFP in budding yeast cells, and that their high-level expression caused cellular defects associated with localization processes. We further show that limitation of the exportin Crm1 determined the expression limit of GFP with a nuclear export signal. Although misfolding of GFP with a vesicle-mediated transport signal triggered endoplasmic reticulum stress, it was not the primary determinant of its expression limit. The precursor of GFP with a mitochondrial targeting signal caused a cellular defect. Finally, we estimated the residual capacities of localization processes. High-level expression of a localized protein thus causes cellular defects by overloading the capacities of localization processes.

Protein turnover requires cellular resources. However, because resources are finite, ultimate high-level expression of a gratuitous protein potentially leads to overloading and exhaustion of resources^1^. Ultimate high-level expression of a gratuitous protein, in fact, monopolizes cellular resources for protein synthesis and causes cellular growth defects^2^,^3^,^4^,^5^,^6^. In addition to synthesis, protein turnover requires cellular resources for folding, degradation, post-translational modification, and localization. High-level expression of a protein imposes a high demand on these resources and potentially overloads them; for example, high-level expression of an aggregative polyQ-containing protein causes cellular growth defects by sequestering and limiting the chaperone Sis1^7^; disomic yeast strains show growth defects because overexpression of proteins from the extra chromosome overloads the degradation machinery, proteasome^8^. High-level expression of yellow fluorescent proteins (YFPs) with misfolding mutations cause cellular growth defects^9^, while a green fluorescent protein (GFP) with a degradation signal has a stronger negative effect on cellular growth than normal GFP^10^. These proteins may also overload folding and degradation resources when they are highly expressed.

For localization of proteins to intracellular compartments, specific types of transport machinery are used. Localization of proteins is usually performed based on the information of localization signals^11^, and the presence of these signals may be predicted based, in part, on their consensus amino acid sequences. Mitochondrial targeting signals (MTSs) and signal sequences (SSs) located at the N termini of proteins are used to target proteins into the mitochondria and the endoplasmic reticulum (ER), respectively^12^,^13^. Nuclear localization signals (NLSs) are used to import proteins into the nucleus^14^, and nuclear export signals (NESs) are used to export proteins from the nucleus^15^. The C termini of some proteins contain cytoplasmic membrane-anchoring signals^16^, and these localization/targeting signals are recognized by specific transport machinery^11^,^17^,^18^,^19^. Because transport machinery is also a limited cellular resource, high-level expression of a transported protein potentially leads to overload of the transporting process, prevents the transport of other essential proteins, and causes cellular growth defects. However, the overload of localization resources and the physiological consequences of this have never been studied experimentally.

The genetic tug of war (gTOW) is a method for estimating the overexpression limit of a protein in yeasts^20^,^21^,^22^. In a gTOW experiment, the limit leading to cellular growth defects is measured as the copy-number limit of the gene encoding the target protein (for details of the gTOW experiment, see Supplementary Method). Previously, we measured the expression limits of a model gratuitous protein, GFP, using the gTOW in the budding yeast *Saccharomyces cerevisiae*^10^. We also observed that addition of a degradation signal to GFP reduced the expression limit, probably due to the overloading of degradation resources^10^. In this study, we attached various localization signals to GFPs and measured their expression limits using the gTOW method. On the basis of these measurements, we evaluated the overloading of localization resources that correspond to various cellular compartments and analyzed the cellular defects triggered by these loads.

## Results

### Localization signals affect expression limits of GFP

**Figure 1:**
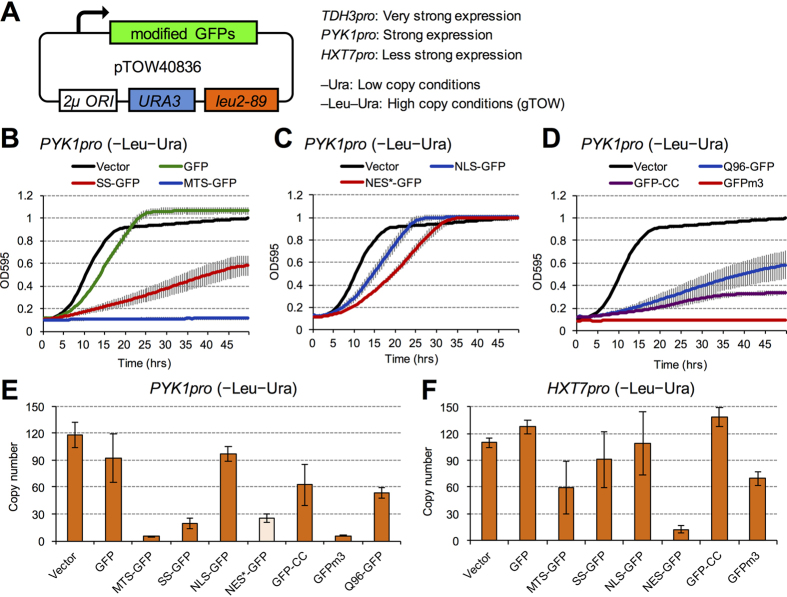
High-level expression of modified GFPs affects cellular growth. (**A**) Structure of the plasmid used in this study. Modified GFPs are expressed from *TDH3pro*, *PYK1pro*, or *HXT7pro*. The copy number of the plasmid is low under –Ura conditions and high under –Leu–Ura conditions, owing to the selection bias imposed by *leu2d* (for details of the gTOW experiment, see Supplementary Method). (**B**–**D**) Growth curve of cells expressing modified GFP under –Leu–Ura conditions. The optical densities at 595 nm (OD_595_) of the cell cultures were measured every 30 minutes. Error bars represent standard deviation. (**E**,**F**) Copy numbers of the gTOW plasmids containing modified GFPs under –Leu–Ura conditions. Modified GFPs were expressed from *PYK1pro* (**E**) or *HXT7pro* (**F**). Error bars represent standard deviations.

To evaluate overloading of localization processes, we attached various localization/targeting signals to GFPs and measured their expression limits using gTOW. We used codon-optimized enhanced GFP (yEGFP3)^23^ as a model gratuitous protein. Herein, we designate yEGFP3 without any additional sequence or mutation as “GFP” unless otherwise stated. We expected that the limit of each localization process will be reflected in a decreased expression limit of the GFP modified with a given signal sequence and that the overload of this process will cause a specific growth defect. We analyzed mitochondrial targeting, vesicular transport, nuclear localization, nuclear export, and cytoplasmic membrane anchoring processes by attaching each corresponding signal sequence to either the N or C terminus of a GFP (MTS-GFP, SS-GFP, NLS-GFP, NES-GFP, and GFP-CC; Table 1). Characterization of the MTS from *S. cerevisiae* Mrps12 is shown in Supplementary Figure S1. We also analyzed a polyglutamine chain attached to a GFP (Q96-GFP), a misfolding GFP (GFPm3), and a proteasome-dependent degron attached to a GFP (GFP-Deg) as reference proteins causing growth defects on high-level expression (Table 1). GFPs and modified GFPs were expressed using a very strong *TDH3* promoter (*TDH3pro*), strong *PYK1/CDC19* promoter (*PYK1pro*), or less strong *HXT7* promoter (*HXT7pro*)^24^, and the genes were cloned into the gTOW plasmid pTOW40836 (Fig. 1A)^22^. Budding yeast BY4741 cells were transformed with these plasmids, and the copy numbers of gTOW plasmids containing the target genes were measured to estimate the expression limits of modified GFPs.

The growth curves of cells expressing modified GFPs from *PYK1pro* under –Leu–Ura conditions are shown in Fig. 1B–D, while the growth rates of cells harboring the gTOW plasmids in –Ura and –Leu–Ura are shown in Supplementary Figure S2. The growth rate of GFP was significantly lower than that of the empty vector (*p* < 1.0E-4), and cells expressing modified GFPs showed further growth retardation (*p* < 1.0E-6). Copy numbers of gTOW plasmids containing modified GFPs expressed from *PYK1pro* under –Leu–Ura conditions are shown in Fig. 1E. Copy-number limits of modified GFPs, with the exception of NLS-GFP, were significantly lower than the copy-number limit of GFP (*p* < 0.05). We could not analyze NES expressed from *PYK1pro*, probably due to its strong negative effect on cellular growth, although we accidentally obtained a NES mutant containing a 3-amino-acid deletion (NES^*^) (Table 1) that caused milder growth defects and used it for further *PYK1pro* experiments. The copy numbers of gTOW plasmids containing modified GFPs expressed from *HXT7pro* in –Leu–Ura are shown in Fig. 1F. As expected, overall copy numbers were higher than those in *PYK1pro* experiments because *HXT7pro* is weaker, but copy-number limits of MTS-GFP, SS-GFP, NES-GFP, and GFPm3 were still significantly lower than the copy-number limit of GFP (*p* < 0.05). Of these, the NES-GFP showed the lowest copy-number limit. These results indicate that high-level expression of GFPs with various localization signals, with the exception of NLS, affected cellular growth and thus reduced the expression limits of GFPs in the same way as the misfolding and aggregative proteins GFPm3 and Q96-GFP.

The expression limits of modified GFPs were independent of their fluorescent activities. We analyzed modified GFPs with mutations in the fluorophore (GFP-Y66G)^25^, and found that the copy-number limits of modified GFP-Y66Gs were indistinguishable from those of modified GFPs (Supplementary Figure S3).

### High-level expression of modified GFPs triggers transcriptional responses associated with modifications and localization signals

**Figure 2:**
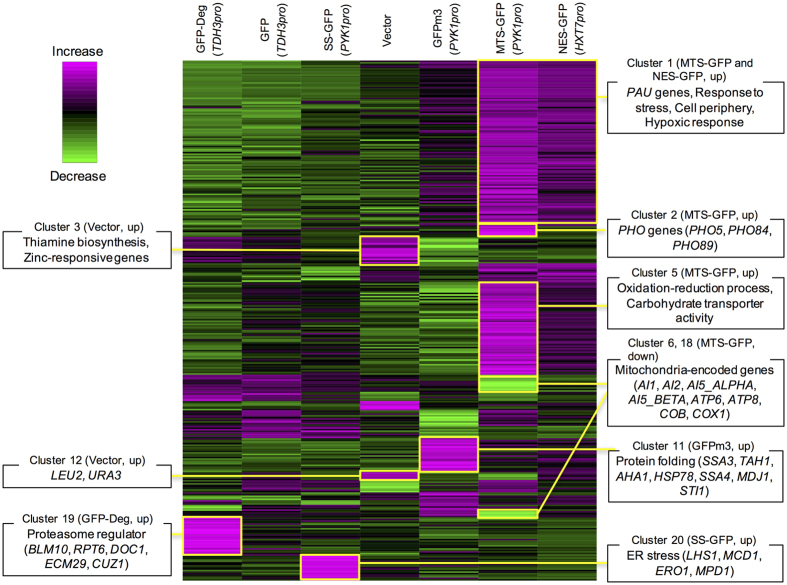
Transcriptional response upon high-level expression of modified GFP. The mRNAs expressed in the cells expressing modified GFPs were analyzed by DNA microarray analysis. Expressed modified GFPs and the promoters are shown at the top. Genes whose expression levels changed by greater than two-fold over the mean of all experiments were isolated and divided into 20 clusters according to the similarities in their expression profiles. Clusters containing genes with specific functional categories are shown. Expression patterns of characteristic clusters are shown in Supplementary Figure S4, and genes in each cluster are listed in Supplementary Table S2.

Then, to elucidate the physiological consequences of the high-level expression of modified GFPs, we performed a transcriptome analysis using an oligo DNA microarray. We analyzed transcripts of cells harboring pTOW40836 plasmids with MTS-GFP (*PYK1pro*), SS-GFP (*PYK1pro*), GFPm3 (*PYK1pro*), NES-GFP (*HXT7pro*), GFP (*TDH3pro*), and GFP-Deg (*TDH3pro*) cultured in –Ura conditions because cells showed growth defects under these conditions (Supplementary Figure S2). We also analyzed an empty vector (pTOW40836) as a control. Figure 2 shows the result of a hierarchical-clustering analysis of 337 genes whose expressions were changed more than two-fold over the average of all experiments, with gene categories enriched in each cluster. Expression patterns of characteristic clusters are shown in Supplementary Figure S4, and genes in each cluster are listed in Supplementary Table S2.

In the empty-vector experiment, expression levels of *LEU2* and *URA3* (cluster 12) were higher than those of other experiments, as reflected in the plasmid copy number. Expression of thiamine biosynthesis and zinc-responsive genes (cluster 3) were also higher, but for unknown reasons. Expression of modified GFPs, with the exception of NES-GFP, induced transcriptional responses associated with their localization signals and modulation. Expression of proteasome regulators (cluster 19) was induced on high-level expression of GFP-Deg. Expression of ER stress-related genes (cluster 20) was induced on high-level expression of SS-GFP. Expression of genes involved in protein folding (cluster 11) was induced on high-level expression of GFPm3, confirming a previous analysis of proteomes^9^. Expression of mitochondria-encoded genes (clusters 6 and 18) was reduced on high-level expression of MTS-GFP, which also induced the expression of genes (cluster 1, 2, and 5) significantly enriched in some functional categories. NES-GFP showed transcriptional responses similar to those of MTS-GFP with respect to clusters 1 and 5, but we could not identify a specific transcriptional response. Transcriptome responses thus support the hypothesis that high-level expression of modified GFPs overloads cellular processes associated with localization signals and modifications.

### Cargo size and a mutation in the exportin Crm1 affect the limits of NES-GFPs

NES-GFP had the lowest expression limit among the tested localization signals (Fig. 1F). Microarray analysis, however, yielded no evidence that high-level expression of NES-GFP causes overloading of nuclear export processes. We considered two possible causes of cellular growth defects when a NES-containing protein is highly expressed. One is process overload, in which high-level expression of a NES-containing protein overloads the transport machinery, and the other is direct toxicity, in which a highly expressed NES peptide nonspecifically binds to unknown essential factors and affects their functions.

**Figure 3:**
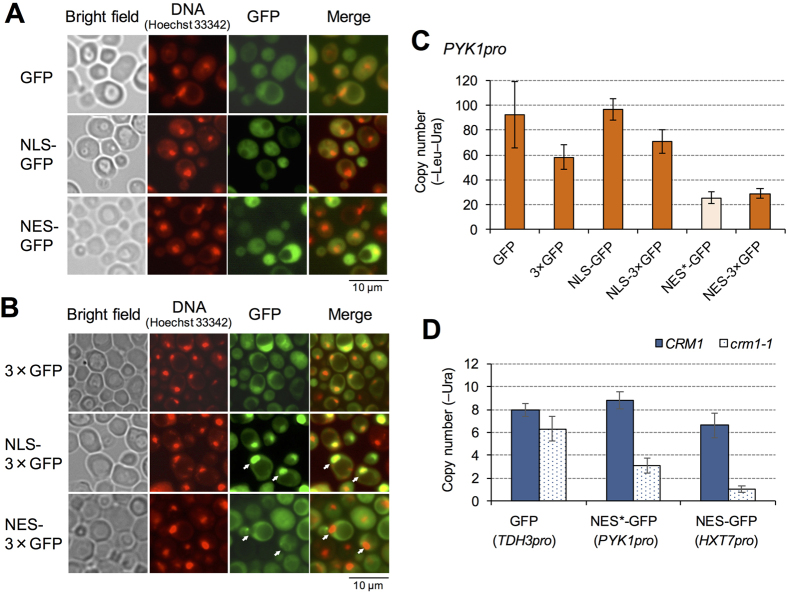
Effect of cargo size and exportin Crm1 mutation on the limits of NES-GFPs. (**A**,**B**) Localization of monomeric GFPs with NLS and NES (**A**) and 3×GFP with NLS and NES (**B**). Cells expressing indicated modified GFPs from *PYK1pro* on gTOW plasmids cultured in –Ura medium were observed. Arrowheads indicate the positions of nuclei. **C**) Copy numbers of gTOW plasmids containing indicated modified GFPs expressed from *PYK1pro* under –Leu–Ura conditions. **D**) Copy numbers of gTOW plasmids containing indicated modified GFPs expressed from indicated promoters within the wild type (*CRM1*) and the *crm1-1* mutant cells cultured at 30 °C in –Ura conditions. Error bars represent standard deviations.

To distinguish these possibilities, we constructed triple GFPs (3×GFP) with NES and NLS. A nuclear pore has an exclusion molecular weight limit, and small proteins such as GFP monomers (27 kDa) diffuse freely into and out of the nucleus^26^. Thus, we increased the size of cargo proteins using 3×GFP (81 kDa) to reduce the speed of free diffusion (our working models are shown in Supplementary Figure S5). Expression of 3×GFPs was validated using Western blot analysis in Supplementary Figure S6, and the functionalities of NLS and NES used here were evaluated in Supplementary Figure S7. Although GFP monomers with NLS and NES were localized throughout the nucleus and cytoplasm (Fig. 3A), 3×GFP with NLS and NES indeed were localized inside and outside the nucleus, respectively (white arrowheads in Fig. 3B). If the transport process is overloaded by the high-level expression of NES-GFP, reducing the diffusion speed using 3×GFP will increase the expression. On the other hand, if the NES peptide shows direct toxicity, cargo size will not change the expression limit. Figure 3C shows the copy-number limits of GFP and 3×GFP with NLS and NES. The copy-number limits of 3×GFP and NLS-3×GFP were lower than those of GFP and NLS-GFP, probably because 3×GFP exerts a higher protein burden effect^6^. In contrast, NES-3×GFP showed a higher copy-number limit than NES-GFP, and in particular, NES (without the 3-amino-acid deletion) could be expressed from *PYK1pro* when 3×GFP was used, whereas only NES^*^ (the mutant NES with the 3-amino-acid deletion) could be analyzed in the case of a single GFP (see above). These results support the hypothesis that high-level expression of NES-GFP overloads the nuclear export process.

The export carrier of the NES used in this study is the exportin Crm1^27^. Thus, it is possible that Crm1 is the primary limiting factor in nuclear export overload when NES-GFP is highly expressed. We accordingly measured the expression limits of NES-GFPs in a temperature-sensitive *crm1* mutant (*crm1-1*), and as shown in Fig. 3D, the copy-number limits of NES-GFP (*HXT7pro*), NES^*^-GFP (*PYK1pro*), and NES-3×GFP (*PYK1pro*) in *crm1-1* mutant cells were significantly reduced compared with those in the wild type cells (*p* < 1.0E-8). This suggests that high-level expression of a NES-containing protein overloads the Crm1-mediated nuclear export process.

### The ER stress triggered by GFP misfolding is not the primary determinant of the expression limit of SS-GFP

**Figure 4:**
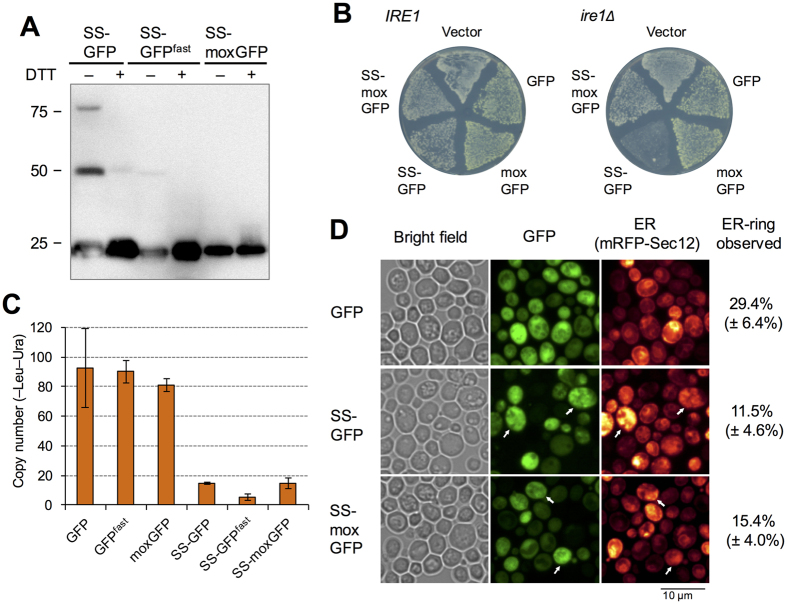
ER stress triggered by misfolding of GFP is not the primary determinant of the expression limit of SS-GFP. (**A**) Western blot analysis of SS-GFPs using anti-GFP antibodies. Protein samples were prepared from cells cultured in –Ura medium. Molecular weight (kDa) is shown on the left. DTT: dithiothreitol. (**B**) Growth of wild type (*IRE1*, BY4741) and *ire1Δ* cells upon high-level expression of modified GFPs. Cells expressing indicated modified GFPs were spread onto –Ura plates. (**C**) Copy numbers of modified GFPs in –Leu–Ura conditions. Error bars represent standard deviations. (**D**) Localization of modified GFPs. Arrowheads indicate aberrant and aggregated ER structures co-localizing with SS-GFP and SS-moxGFP. Percentages of normal two-ring ER structures observed with mRFP-Sec12 are also shown.

Commonly used GFPs are poorly folded and are misfolded into disulfide-linked oligomers in the ER^28^,^29^. Thus, we speculate that the misfolded GFP triggers the ER stress response that we observed upon high-level expression of SS-GFP (Fig. 2) and that the expression limit of SS-GFP is determined by overload of the ER protein quality-control machinery. We accordingly tested a recently developed moxGFP, which lacks cysteine and does not misfold in the ER^29^, as well as GFP^fast^, which folds faster and avoids misfolding in the ER^28^. Using Western blot analysis, we confirmed that SS-GFP constitutes disulfide bond-linked oligomers but that SS-GFP^fast^ and SS-moxGFP do not (Fig. 4A). Next, we tested whether the defects in the ER stress response affect the expression limits of SS-GFP. As shown in Fig. 4B, cells with a deletion of *IRE1*, an essential factor in ER stress response, showed severe growth defects upon high-level expression of SS-GFP but not in the case of SS-moxGFP. This result supports the idea that the expression limits of SS-GFP are determined by overload of the ER quality control machinery. However, the copy-number limit of SS-moxGFP was still as low as that of SS-GFP, and the limit of SS-GFP^fast^ was even lower (Fig. 4C). Then, we observed the ER structure of cells expressing high-level SS-GFP and SS-moxGFP, as shown in Fig. 4D. In the budding yeast, ERs are usually observed as two-ring structures (a nuclear envelope and a cortical ER)^30^. When SS-GFP is highly expressed, they sometimes showed aberrant, aggregated structures that co-localize with SS-GFP, and changing GFP into moxGFP did not revert these aberrant ER structures. These results suggest that high-level expression of SS-GFP and SS-moxGFP affects normal ER functions. We thus concluded that the ER stress triggered by GFP misfolding is not the primary determinant of the SS-GFP expression limit.

### Mislocalization-triggered cellular defects may determine the low expression limit of MTS-GFP

**Figure 5:**
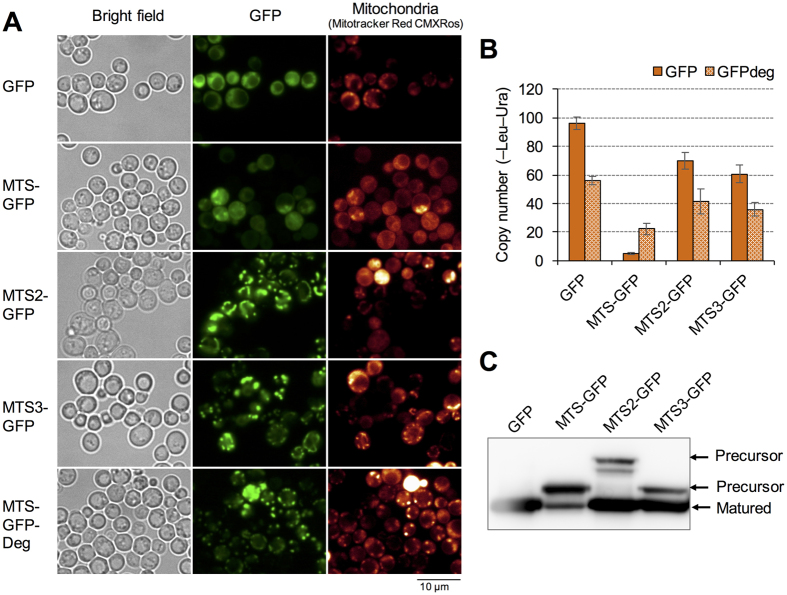
Growth defects triggered by a mislocalized cytoplasmic precursor may determine the expression limit of MTS-GFP. (**A**) Localization of MTS-GFPs. Cells expressing indicated modified GFPs from *PYK1pro* on gTOW plasmids cultured in –Ura medium were observed. (**B**) Copy numbers of gTOW plasmids containing indicated modified GFPs under –Leu–Ura conditions. (**C**) Western blot analysis of MTS-GFPs using anti-GFP antibodies. Protein samples were prepared from cells cultured in –Ura medium. Precursors and matured form of MTS-GFPs are indicated. Uncropped original blot image can be found in Supplementary Figure S12.

The transcriptome analysis above suggests that high-level expression of MTS-GFP causes mitochondrial defects (Fig. 2). Figure 5A shows a microscopic observation of MTS-GFP. We observed a few normal mitochondrial structures as seen in the GFP control in cells expressing high-levels of MTS-GFP. MTS-GFP was observed throughout the cytoplasm and cell periphery, sometimes as dots; it has been reported that GFP with MTS from *Neurospora crassa* Su9 protein (MTS2, Table 1) localized to the mitochondria even when highly expressed from the *ADH1* promoter on a 2-μm plasmid^31^. Thus, we accordingly analyzed the expression limit of MTS2-GFP. We also analyzed a MTS from *S. cerevisiae* Adh3 (MTS3, Table 1) because this is one of the highly expressed mitochondrial proteins^32^,^33^. Interestingly, MTS2-GFP and MTS3-GFP have much higher copy-number limits than MTS-GFP (Fig. 5B); MTS2-GFP and MTS3-GFP localized to the mitochondria even under the same conditions when MTS-GFP mislocalized (*PYK1pro*, –Ura). MTSs are usually removed after transport into the mitochondria by matrix proteases^17^; nevertheless, we found that some fraction of MTS-GFP, MTS2-GFP, and MTS3-GFP remained as precursors under our experimental conditions (arrowheads in Fig. 5C). Consistent with this localization pattern, non-transported MTS-GFP precursors were much more abundant than the matured form. From these results, we hypothesize two alternative causes for cellular defects when MTS-GFP is highly expressed; MTS-GFP overloads mitochondrial transport machinery with lower amounts than MTS2 and MTS3, or the precursor form of MTS-GFP causes cellular defects, probably due to the properties of the MTS amino acid sequence (our working hypothesis is shown in Supplementary Figure S8A). To test these hypotheses, we added a proteasome-dependent degron (Deg, see above) to MTS-GFP to selectively digest the cytoplasmic precursor of MTS-GFP (our working hypothesis is shown in Supplementary Figure S8B, and reduction of the precursor form in MTS-GFP-Deg was confirmed by Western blot analysis as shown in Supplementary Figure S9). Interestingly, MTS-GFP-Deg clearly localized to the mitochondria and did not cause an aberrant mitochondrial morphology (Fig. 5A). In addition, MTS-GFP-Deg had a higher copy-number limit than MTS-GFP, although the attachment of Deg to GFP, MTS2-GFP, and MTS3-GFP reduced this limit, probably due to degradation overload (Fig. 5B). This result supports the idea that mislocalized MTS-GFP precursors cause cellular defects, including mitochondrial dysfunction, and limit its expression. In addition, it has been suggested that effective MTSs, like MTS2 and MTS3, transport GFPs without strong cellular defects.

### Estimation of protein expression limits of GFPs with localization signals

**Figure 6:**
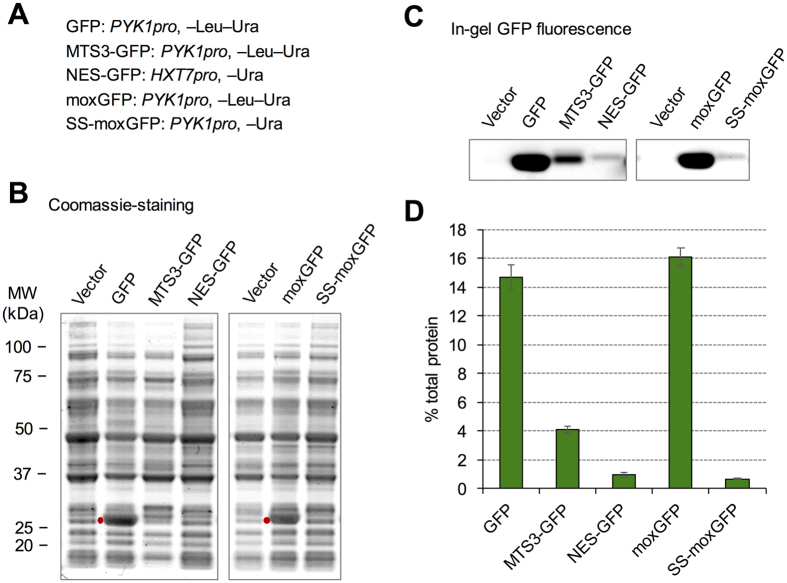
Estimation of protein expression limits of GFPs with localization signals. (**A**) Promoter and growth conditions used to express each modified GFP. (**B**) Coomassie staining of SDS-PAGE-separated total cellular proteins. Red points indicate GFP bands. (**C**). In-gel fluorescence of modified GFPs. Uncropped original blot images can be found in Supplementary Figure S12. (**D**) Expression levels of modified GFPs as a proportion of total protein.

We next estimated the protein amount sufficient to cause growth defects due to the overload of each localization process by measuring the amounts of GFPs with localization signals. As shown in Fig. 6A, we measured the amounts of MTS3-GFP (*PYK1pro*, –Leu–Ura), NES-GFP (*HXT7pro*, –Ura), and SS-moxGFP (*PYK1pro*, –Ura) as we considered them to be expressed to the upper limits of mitochondrial targeting, nuclear export, and vesicle-mediated transport, respectively. The amounts were then compared with those of GFP (*PYK1pro*, –Leu–Ura) and moxGFP (*PYK1pro*, –Leu–Ura), which reflect total cytoplasmic protein production capacity. We found that GFP and moxGFP could be recognized as bold bands among the total proteins separated by SDS-PAGE (red points in Fig. 6B) and accordingly first estimated the GFP levels as percentage protein with respect to total protein by measuring the intensity of GFP bands and then divided them by the total protein intensity after protein fluorescent labeling (Table 2; calculation method is shown in Supplementary Figure S10). We then measured the protein levels using the in-gel GFP fluorescence of SDS-PAGE-separated total cellular protein (Fig. 6C). Estimated protein expression limits of mitochondrial transport, nuclear export, and vesicle-mediated transport were 4.1%, 1.0%, and 0.7% of total protein, respectively (Fig. 6D, Table 2).

## Discussion

In this study, we aimed to evaluate the overloads of protein localization processes by measuring expression limits of GFPs with various localization signals using the gTOW method. Attachment of localization signals tested in this study, with the exception of NLS, reduced the copy-number limit of GFP (Fig. 1), suggesting that high-level expression of proteins with localization signals cause growth defects, just as is the case for misfolded and aggregative proteins. Transcriptome analysis suggested that high-level expression of localization signal-attached GFPs causes defects in their targeted cellular processes (Fig. 2). Among the localization signals tested in this study, NES-GFP had the lowest expression limit (Fig. 1). The finding that expression limit was further reduced in exportin mutant *crm1-1* cells suggested that high-level NES-GFP triggers the exhaustion of Crm1 (Fig. 3D). In contrast, high-level expression of NLS-GFP did not show a low expression limit like that of NES-GFP (Fig. 1). Thus, the nuclear–cytoplasmic transport process may have asymmetric capacity. We found no evidence that transport factors are exhausted when MTS-GFP or SS-GFP are highly expressed; limiting factors might be identified by the systematic screening of mutants where expression limits are reduced. “Localization processes” described here can be dissected into individual resource-consuming processes, such as recognition and modification of localization signals, transportation, response against stresses caused by localization signals, and removal of mislocalized proteins, and so on. (Supplementary Figure S11). Factors involved in these individual processes could be the limiting factors to be identified.

The expression limits of GFPs with localization signals are affected by size and folding properties of cargo proteins (Figs 3 and 4) and by the properties of the localization signal (Fig. 5). The reason behind the low expression limit of NES-GFP may be the use of monomeric GFP, which is smaller than the exclusion limit of the nuclear pore and freely diffuses through it; indeed, increasing cargo size by using 3×GFP increased the expression limit (Fig. 3C). Thus, it is possible that amino acid sequences matching the consensus sequences of the nuclear export signal could be avoided in a small protein because they readily overload the nuclear–cytoplasmic transport process. It is known that the slowly folding proteins, including GFP, which we used first in this study, sometimes misfold in the ER^28^,^29^. Indeed, we found that high-level expression of SS-GFP induced the ER stress response (Fig. 2) and led to misfolding (Fig. 4A). However, SS-moxGFP, which avoids misfolding^29^, had the same expression limit as SS-GFP (Fig. 4C), indicating that GFP misfolding is not the primary determinant of the expression limit of SS-GFP. The rapidly folding proteins, such as dihydrofolate reductase and GFP, could trigger “clogging” of the transport process in the ER^34^, which may be another factor limiting the expression of SS-GFPs. MTS (MTS from Mrps12, Supplementary Figure S1), which we used in the initial analysis, showed a relatively low expression limit (Fig. 1). Accumulation of the precursor of MTS-GFP in the cytoplasm appeared to account for the low limit, given that removal of the cytoplasmic precursor increased the expression limit (Fig. 5B). Other MTSs, such as MTS2 and MTS3, were effectively transported into mitochondria (Fig. 5A), and their expression limits were much higher (Fig. 5B). These observations suggest that high-level expression of mitochondrial proteins with ineffective MTSs causes cellular growth defects due to negative effects of the precursors. Cytoplasmic accumulation of precursors of mitochondrial proteins has recently been reported to trigger proteostatic stress^35^,^36^. In this study, we showed that artificial attachment of a proteasome-dependent degradation signal increased the expression limit of MTS-GFP (Fig. 5B). Intrinsic mitochondrial proteins whose precursors have negative effects in the cytoplasm may be removed by the proteasome.

In this study, we measured the protein expression limits of GFPs with localization signals (Fig. 6). Although the limit of GFP was 15% of total protein, the limits of MTS-GFP, NES-GFP, and SS-GFP were restricted to 4%, 1%, and 0.7%, respectively (Table 2 and Fig. 6). We propose that this restriction reflects the capacity of each transport process and potentially causes a growth defect upon overexpression of endogenous proteins with localization signals. We note that we estimated protein expression limits out of total protein. Estimating the protein expression limit for each intracellular compartment using fractionated organelles might help to precisely understand the capacity. In eukaryotic cells, over half of proteins localize to an intracellular compartment^37^. Because the amount of a target protein expressed in overexpression experiments reaches approximately 1% of total protein^38^, a large proportion of proteins that cause growth defects upon overexpression probably comprise proteins triggering process overloads. Aneuploid cells such as cancer cells in which many proteins are simultaneously overexpressed are expected to suffer from process overloads, and the expression of heterologous proteins is restricted by the capacities of transport processes. Thus, understanding and controlling process overloads is beneficial for disease treatment and cellular engineering.

## Materials and Methods

### Strains, growth conditions, and yeast transformation

BY4741 (*MATa his3Δ1 leu2Δ0 met15Δ0 ura3Δ0*)^39^ was used as the host strain for the experiments. The *ire1Δ* (*MATa ire1Δ*::*KanR his3Δ1 leu2Δ0 met15Δ0 ura3Δ0*) and *crm1-1* (*MATa crm1-1*::*KanR his3Δ1 leu2Δ0 met15Δ0 ura3Δ0*) strains were derivatives of BY4741^40^,^41^. Yeast culture and transformation were performed as previously described^42^. Synthetic complete (SC) medium without uracil (Ura) and leucine (Leu) as indicated were used for yeast culture.

### Plasmids used in this study

The plasmids used in this study are listed in Supplementary Table S3. The plasmids were constructed on the basis of the homologous recombination activity of yeast cells^43^, and their sequences were verified by DNA sequencing. Files for the ApE software containing the DNA sequences and annotations of plasmids are provided upon request. The mRFP-SEC12 plasmid (pRS413-mRFP-SEC12) was constructed by transferring the *TDH3pro-mRFP-SEC12* fragment from 316-mRFP-Sec12^44^ into pRS413^45^.

### Measurement of plasmid copy-number limit

DNA from yeast cells grown in either a SC–Ura or a SC–Leu–Ura medium was prepared according to a previously described method^20^. The plasmid copy number was measured by real-time PCR as previously described^20^ using a Lightcycler480 (Roche). *LEU2* primer set (LEU2-2F: 5′-GCTAATGTTTTGGCCTCTTC-3′; LEU2-2R: 5′-ATTTAGGTGGGTTGGGTTCT-3′) and *LEU3* primer set (LEU3-3F: 5′-CAGCAACTAAGGACAAGG-3′; LEU3-3R: 5′-GGTCGTTAATGAGCTTCC-3′) were used to amplify DNA fragments of the pTOW40836 plasmid and genomic DNA, respectively. Average values, standard deviation (SD), and *p*-value of Student’s *t* tests were calculated from at least three independent biological experiments.

### Measuring maximum growth rate and GFP fluorescence

Cellular growth was measured by monitoring OD_595_ every 30 minutes using an Infinite F200 microplate reader (TECAN). The maximum growth rate (MGR) was calculated as described previously^20^. Average values, standard deviation (SD), and *p*-values of Student’s *t* test were calculated from at least three independent biological experiments.

### Microarray analysis

Total RNA was extracted from log-phase cells cultured in SC–Ura medium using the hot-phenol method^46^. Equipment and reagents for the subsequent microarray analysis were provided by Agilent Technologies unless otherwise stated. Quality of the RNA was checked using the 2100 bioanalyzer. The RNA was labeled using the Low Input Quick Amp Labeling Kit One-Color, hybridized to the Yeast oligo DNA microarray version 2.0 using a Gene Expression Hybridization Kit, and washed using the Gene Expression Wash Buffers Pack as described in the manufacturer’s protocol. The hybridized mRNAs were scanned and quantified using the SureScan microarray scanner and the Feature Extraction software. The raw data were processed using the agilp R-Bioconductor package (https://www.bioconductor.org/packages/release/bioc/html/agilp.html). We extracted genes whose expression was changed more than two-fold over the average of all seven experiments. The genes were clustered into 20 clusters by the hierarchical-clustering (average) method using R (https://www.r-project.org). Gene ontology and publication significantly enrich the genes were surveyed using the List Analysis of YeastMine (http://yeastmine.yeastgenome.org/). All raw data was deposited in the GEO database (http://www.ncbi.nlm.nih.gov/geo/) under accession number GSE76080.

### Microscopic observation

Log phase cells were cultivated in a SC–Ura medium. Cellular images were obtained and processed using the DMI6000 B microscope and Leica Application Suite X software (Leica Microsystems). GFP fluorescence was observed using the GFP filter cube. Cellular DNA was stained with 100 μg/ml Hoechst 33342 (H3570, ThermoFisher) for 5 min and observed using the A filter cube. The mitochondrion was stained with 50 ng/ml MitoTracker Red CMXRos (M7512, ThermoFisher) for 30 minutes and observed using the RFP filter cube. The ER was observed using mRFP-Sec12 using the RFP filter cube. Percentages of normal two-ring ER structures observed with mRFP-Sec12 were counted from approximately 300 cells. The mean and standard deviation (SD) were calculated from five independent images.

### Protein analysis

Total protein was extracted from log-phase cells with NuPAGE LDS sample buffer (ThermoFisher NP0007) after 0.2N NaOH treatment^47^. For each analysis, total protein extracted from 100 μL of cells with OD_600_ 1.0 was used. For total protein visualization, the extracted total protein was separated by SDS-PAGE and stained by Coomassie staining (LC6060, ThermoFisher). For detection of GFP, the SDS-PAGE separated proteins were transferred to a PVDF membrane. GFP was detected using an anti-GFP antibody (11814460001, Roche), a peroxidase-conjugated second antibody (414151F, Nichirei Biosciences), and a chemiluminescent regent (34095, ThermoFisher). The chemiluminescent image was acquired with an LAS-4000 image analyzer in chemiluminescence detection mode (GE Healthcare). In-gel GFP fluorescence was measured as follows: SDS-PAGE-separated GFP was refolded by soaking in phosphate-buffered saline (P4417, Sigma) with 0.1% Tween 20 for two hours and then GFP fluorescence was detected and measured using the LAS-4000 image analyzer in GFP fluorescence detection mode and Image Quant TL software (GE Healthcare). For estimation of GFP amount as a proportion of total protein, the extracted total protein was labeled with Ezlabel FluoroNeo (WSE-7010, Atto) as described in the manufacturer’s protocol and separated by SDS-PAGE. Proteins were detected and measured using the LAS-4000 image analyzer in SYBR-Green fluorescence detection mode and Image Quant TL software. The GFP expression level was calculated as described in Supplementary Figure S10. Average values, standard deviation (SD), and *p*-values of Student’s *t* test were calculated from at least three independent biological experiments.

## Additional Information

**How to cite this article**: Kintaka, R. *et al*. Cellular growth defects triggered by an overload of protein localization processes. *Sci. Rep.* **6**, 31774; doi: 10.1038/srep31774 (2016).

## Supplementary Material

Supplementary Information

Supplementary Table S1

Supplementary Table S2

Supplementary Table S3

## Figures and Tables

**Table 1:** Localization signals and modifications used in this study.

Name	Origin	Length (a.a.)*	N or C terminal	Reference
Mitochondrial targeting signal (MTS)	Mitochondrial ribosomal protein Mrps12, *S. cerevisiae*	29	N	48
Mitochondrial targeting signal 2 (MTS2)	ATP synthase protein 9, mitochondrial precursor *N. crassa*	69	N	31
Mitochondrial targeting signal 3 (MTS3)	Mitochondrial alcohol dehydrogenase Adh3, *S. cerevisiae*	29	N	33
Signal sequence for vesicle-mediated transport (SS)	EP procyclin, *T. brucei*	29	N	49,50
Nuclear localization signal (NLS)	Large T-antigen, SV40	8	N	51,52
Nuclear export signal (NES)	cAMP-dependent protein kinase inhibitor, *H. sapiens*	15	N	51,53
Nuclear export signal short (NES*)	cAMP-dependent protein kinase inhibitor, *H. sapiens*	12	N	This study
Cytoplasmic membrane retention signal (CC)	Casein kinase Yck2, *S. cerevisiae*	186	C	54
Poly glutamine stretch (Q96)	huntingtin exon 1, *H. sapiens*	183	N	7
Misfolding mutant (m3)	Synthetic (N23I, E32K, G40V)	—	—	9
Degradation signal (Deg)	Ornithine decarboxylase, *M. musculus*	25	C	55

^*^Amino acid sequences are listed in Supplementary Table S1.

**Table 2:** Estimated protein expression limits of modified GFPs.

Expressed protein (promoter, conditions)	% total protein (A)	% GFP (B)	A × B ÷ 100 (% total protein)
GFP (*PYK1pro*, –Leu–Ura)	14.7 ± 0.7	100.0 ± 5.6	14.7 ± 0.8
MTS3-GFP (*PYK1pro*, –Leu–Ura)		28.1 ± 1.6	4.1 ± 0.2
NES-GFP (*HXT7pro*, –Ura)		6.7 ± 1.0	1.0 ± 0.1
moxGFP (*PYK1pro*, –Leu–Ura)	16.1 ± 0.9	100.0 ± 3.9	16.1 ± 0.6
SS-moxGFP (*PYK1pro*, –Ura)		4.6 ± 0.1	0.7 ± 0.0

Values are mean ± standard deviation.

## References

[b1] Moriya H. Quantitative nature of overexpression experiments. Mol Biol Cell 26, 3932–3939, 10.1091/mbc.E15-07-0512 (2015). 26543202 PMC4710226

[b2] Snoep J. L., Yomano L. P., Westerhoff H. V. & Ingram L. O. Protein burden in *Zymomonas rnobilis*: negative flux and growth control due to overproduction of glycolytic enzymes. Microbiology 141, 2329–2337 (1995).

[b3] Stoebel D. M., Dean A. M. & Dykhuizen D. E. The cost of expression of *Escherichia coli lac* operon proteins is in the process, not in the products. Genetics 178, 1653–1660, 10.1534/genetics.107.085399 (2008). 18245823 PMC2278061

[b4] Shachrai I., Zaslaver A., Alon U. & Dekel E. Cost of unneeded proteins in *E. coli* is reduced after several generations in exponential growth. Mol Cell 38, 758–767, 10.1016/j.molcel.2010.04.015 (2010). 20434381

[b5] Shah P., Ding Y., Niemczyk M., Kudla G. & Plotkin J. B. Rate-limiting steps in yeast protein translation. Cell 153, 1589–1601, 10.1016/j.cell.2013.05.049 (2013). 23791185 PMC3694300

[b6] Kafri M., Metzl-Raz E., Jona G. & Barkai N. The Cost of Protein Production. Cell Rep 14, 22–31, 10.1016/j.celrep.2015.12.015 (2016). 26725116 PMC4709330

[b7] Park S. H. . PolyQ proteins interfere with nuclear degradation of cytosolic proteins by sequestering the Sis1p chaperone. Cell 154, 134–145, 10.1016/j.cell.2013.06.003 (2013). 23791384

[b8] Torres E. M. . Identification of aneuploidy-tolerating mutations. Cell 143, 71–83, 10.1016/j.cell.2010.08.038 (2010). 20850176 PMC2993244

[b9] Geiler-Samerotte K. A. . Misfolded proteins impose a dosage-dependent fitness cost and trigger a cytosolic unfolded protein response in yeast. Proc Natl Acad Sci USA 108, 680–685, 10.1073/pnas.1017570108 (2011). 21187411 PMC3021021

[b10] Makanae K., Kintaka R., Makino T., Kitano H. & Moriya H. Identification of dosage-sensitive genes in *Saccharomyces cerevisiae* using the genetic tug-of-war method. Genome Research 23, 300–311, 10.1101/gr.146662.112 (2013). 23275495 PMC3561871

[b11] Matlin K. S. Spatial expression of the genome: the signal hypothesis at forty. Nat Rev Mol Cell Biol 12, 333–340, 10.1038/nrm3105 (2011). 21487438

[b12] Fukasawa Y. . MitoFates: improved prediction of mitochondrial targeting sequences and their cleavage sites. Mol Cell Proteomics 14, 1113–1126, 10.1074/mcp.M114.043083 (2015). 25670805 PMC4390256

[b13] Petersen T. N., Brunak S., von Heijne G. & Nielsen H. SignalP 4.0: discriminating signal peptides from transmembrane regions. Nat Methods 8, 785–786, 10.1038/nmeth.1701 (2011). 21959131

[b14] Kosugi S., Hasebe M., Tomita M. & Yanagawa H. Systematic identification of cell cycle-dependent yeast nucleocytoplasmic shuttling proteins by prediction of composite motifs. Proc Natl Acad Sci USA 106, 10171–10176, 10.1073/pnas.0900604106 (2009). 19520826 PMC2695404

[b15] Xu D. . LocNES: a computational tool for locating classical NESs in CRM1 cargo proteins. Bioinformatics 31, 1357–1365, 10.1093/bioinformatics/btu826 (2015). 25515756 PMC4410651

[b16] Hentschel A., Zahedi R. P. & Ahrends R. Protein lipid modifications-More than just a greasy ballast. Proteomics 16, 759–782, 10.1002/pmic.201500353 (2016). 26683279

[b17] Schmidt O., Pfanner N. & Meisinger C. Mitochondrial protein import: from proteomics to functional mechanisms. Nat Rev Mol Cell Biol 11, 655–667, 10.1038/nrm2959 (2010). 20729931

[b18] Stewart M. Molecular mechanism of the nuclear protein import cycle. Nat Rev Mol Cell Biol 8, 195–208, 10.1038/nrm2114 (2007). 17287812

[b19] Fukata Y. & Fukata M. Protein palmitoylation in neuronal development and synaptic plasticity. Nat Rev Neurosci 11, 161–175, 10.1038/nrn2788 (2010). 20168314

[b20] Moriya H., Shimizu-Yoshida Y. & Kitano H. *In vivo* robustness analysis of cell division cycle genes in *Saccharomyces cerevisiae*. Plos Genetics 2, 1034–1045, 10.1371/journal.pgen.0020111 (2006). PMC1500812 16839182

[b21] Moriya H., Chino A., Kapuy O., Csikasz-Nagy A. & Novak B. Overexpression limits of fission yeast cell-cycle regulators *in vivo* and *in silico*. Molecular Systems Biology 7, 10.1038/msb.2011.91 (2011). PMC3737731 22146300

[b22] Moriya H., Makanae K., Watanabe K., Chino A. & Shimizu-Yoshida Y. Robustness analysis of cellular systems using the genetic tug-of-war method. Molecular Biosystems 8, 2513–2522, 10.1039/c2mb25100k (2012). 22722869

[b23] Cormack B. P. . Yeast-enhanced green fluorescent protein (yEGFP) a reporter of gene expression in *Candida albicans*. Microbiology 143 (Pt 2), 303–311 (1997). 9043107 10.1099/00221287-143-2-303

[b24] Partow S., Siewers V., Bjørn S., Nielsen J. & Maury J. Characterization of different promoters for designing a new expression vector in *Saccharomyces cerevisiae*. Yeast 27, 955–964, 10.1002/yea.1806 (2010). 20625983

[b25] Zacharias D. A. & Tsien R. Y. Molecular biology and mutation of green fluorescent protein. Methods Biochem Anal 47, 83–120 (2006). 16335711

[b26] Popken P., Ghavami A., Onck P. R., Poolman B. & Veenhoff L. M. Size-dependent leak of soluble and membrane proteins through the yeast nuclear pore complex. Mol Biol Cell 26, 1386–1394, 10.1091/mbc.E14-07-1175 (2015). 25631821 PMC4454183

[b27] Güttler T. . NES consensus redefined by structures of PKI-type and Rev-type nuclear export signals bound to CRM1. Nat Struct Mol Biol 17, 1367–1376, 10.1038/nsmb.1931 (2010). 20972448

[b28] Xu C., Wang S., Thibault G. & Ng D. T. Futile protein folding cycles in the ER are terminated by the unfolded protein O-mannosylation pathway. Science 340, 978–981, 10.1126/science.1234055 (2013). 23704572

[b29] Costantini L. M. . A palette of fluorescent proteins optimized for diverse cellular environments. Nat Commun 6, 7670, 10.1038/ncomms8670 (2015). 26158227 PMC4499870

[b30] Du Y., Ferro-Novick S. & Novick P. Dynamics and inheritance of the endoplasmic reticulum. J Cell Sci 117, 2871–2878, 10.1242/jcs.01286 (2004). 15197242

[b31] Westermann B. & Neupert W. Mitochondria-targeted green fluorescent proteins: convenient tools for the study of organelle biogenesis in Saccharomyces cerevisiae. Yeast 16, 1421–1427 (2000). 11054823 10.1002/1097-0061(200011)16:15<1421::AID-YEA624>3.0.CO;2-U

[b32] Kulak N. A., Pichler G., Paron I., Nagaraj N. & Mann M. Minimal, encapsulated proteomic-sample processing applied to copy-number estimation in eukaryotic cells. Nat Methods 11, 319–324, 10.1038/nmeth.2834 (2014). 24487582

[b33] Young E. T. & Pilgrim D. Isolation and DNA sequence of *ADH3*, a nuclear gene encoding the mitochondrial isozyme of alcohol dehydrogenase in Saccharomyces cerevisiae. Mol Cell Biol 5, 3024–3034 (1985). 2943982 10.1128/mcb.5.11.3024-3034.1985 PMC369115

[b34] Ast T., Michaelis S. & Schuldiner M. The Protease Ste24 Clears Clogged Translocons. Cell 164, 103–114, 10.1016/j.cell.2015.11.053 (2016). 26771486 PMC4715265

[b35] Wrobel L. . Mistargeted mitochondrial proteins activate a proteostatic response in the cytosol. Nature 524, 485–488, 10.1038/nature14951 (2015). 26245374

[b36] Wang X. & Chen X. J. A cytosolic network suppressing mitochondria-mediated proteostatic stress and cell death. Nature 524, 481–484, 10.1038/nature14859 (2015). 26192197 PMC4582408

[b37] Chong Y. T. . Yeast Proteome Dynamics from Single Cell Imaging and Automated Analysis. Cell 161, 1413–1424, 10.1016/j.cell.2015.04.051 (2015). 26046442

[b38] Tomala K. & Korona R. Evaluating the fitness cost of protein expression in *Saccharomyces cerevisiae*. Genome Biol Evol 5, 2051–2060, 10.1093/gbe/evt154 (2013). 24128940 PMC3845635

[b39] Brachmann C. B. . Designer deletion strains derived from *Saccharomyces cerevisiae* S288C: a useful set of strains and plasmids for PCR-mediated gene disruption and other applications. Yeast 14, 115–132 (1998). 9483801 10.1002/(SICI)1097-0061(19980130)14:2<115::AID-YEA204>3.0.CO;2-2

[b40] Li Z. . Systematic exploration of essential yeast gene function with temperature-sensitive mutants. Nat Biotechnol 29, 361–367, 10.1038/nbt.1832 (2011). 21441928 PMC3286520

[b41] Giaever G. . Functional profiling of the *Saccharomyces cerevisiae* genome. Nature 418, 387–391, 10.1038/nature00935 (2002). 12140549

[b42] Amberg D. C., Burke D. & Strathern J. N. Methods in Yeast Genetics: A Cold Spring Harbor Laboratory Course Manual. (Cold Spring Harbor Laboratory Press, 2005).

[b43] Oldenburg K. R., Vo K. T., Michaelis S. & Paddon C. Recombination-mediated PCR-directed plasmid construction *in vivo* in yeast. Nucleic Acids Res 25, 451–452 (1997). 9016579 10.1093/nar/25.2.451 PMC146432

[b44] Sato K., Sato M. & Nakano A. Rer1p, a retrieval receptor for ER membrane proteins, recognizes transmembrane domains in multiple modes. Mol Biol Cell 14, 3605–3616, 10.1091/mbc.E02-12-0777 (2003). 12972550 PMC196553

[b45] Christianson T. W., Sikorski R. S., Dante M., Shero J. H. & Hieter P. Multifunctional yeast high-copy-number shuttle vectors. Gene 110, 119–122 (1992). 1544568 10.1016/0378-1119(92)90454-w

[b46] Köhrer K. & Domdey H. Preparation of high molecular weight RNA. Methods Enzymol 194, 398–405 (1991). 1706459 10.1016/0076-6879(91)94030-g

[b47] Kushnirov V. V. Rapid and reliable protein extraction from yeast. Yeast 16, 857–860 (2000). 10861908 10.1002/1097-0061(20000630)16:9<857::AID-YEA561>3.0.CO;2-B

[b48] Fujita K., Horie T. & Isono K. Cross-genomic analysis of the translational systems of various organisms. J Ind Microbiol Biotechnol 27, 163–169 (2001). 11780787 10.1038/sj.jim.7000093

[b49] Clayton C. E. & Mowatt M. R. The procyclic acidic repetitive proteins of *Trypanosoma brucei*. Purification and post-translational modification. J Biol Chem 264, 15088–15093 (1989). 2475493

[b50] Ho H. H., He C. Y., de Graffenried C. L., Murrells L. J. & Warren G. Ordered assembly of the duplicating Golgi in *Trypanosoma brucei*. Proc Natl Acad Sci USA 103, 7676–7681, 10.1073/pnas.0602595103 (2006). 16672362 PMC1472504

[b51] Miller M. E. & Cross F. R. Distinct subcellular localization patterns contribute to functional specificity of the Cln2 and Cln3 cyclins of *Saccharomyces cerevisiae*. Mol Cell Biol 20, 542–555 (2000). 10611233 10.1128/mcb.20.2.542-555.2000 PMC85127

[b52] Kalderon D., Roberts B. L., Richardson W. D. & Smith A. E. A short amino acid sequence able to specify nuclear location. Cell 39, 499–509 (1984). 6096007 10.1016/0092-8674(84)90457-4

[b53] Wen W., Meinkoth J. L., Tsien R. Y. & Taylor S. S. Identification of a signal for rapid export of proteins from the nucleus. Cell 82, 463–473 (1995). 7634336 10.1016/0092-8674(95)90435-2

[b54] Babu P. . Plasma membrane localization of the Yck2p yeast casein kinase 1 isoform requires the C-terminal extension and secretory pathway function. J Cell Sci 115, 4957–4968 (2002). 12432082 10.1242/jcs.00203

[b55] Jungbluth M., Renicke C. & Taxis C. Targeted protein depletion in *Saccharomyces cerevisiae* by activation of a bidirectional degron. BMC Syst Biol 4, 176, 10.1186/1752-0509-4-176 (2010). 21190544 PMC3024245

