# Supplementary material for: Cellular growth defects triggered by an overload of protein localization processes
Source: Sci Rep. 2016 Aug 19;6:31774. doi: 10.1038/srep31774 (PMC4990933; doi:10.1038/srep31774)
Supplement: Supplementary Information [file srep31774-s1.pdf]

Supplementary Information for “Cellular growth defects triggered by an overload of protein localization processes” by Reiko Kintaka, Koji Makanae, and Hisao Moriya

Supplementary Method

gTOW experiment

In gTOW experiments, two conditions were imposed[1,2]: low-copy conditions (–Ura conditions) where average plasmid copy number per cell is approximately 35 owing to *2-μ* plasmid origin on the gTOW plasmids; and high-copy conditions (–Leu–Ura conditions) where the average plasmid copy number of the empty vector reaches >100 per cell owing to the bias of *leu2-89* (a weakly expressed *LEU2* allele) on the gTOW plasmids (Figure 1A). The protein level expressed from the target gene is expected to increase with copy number. If the gene has an overexpression limit of <100 copies, the target gene imposes a selection bias decreasing the plasmid copy number. In gTOW experiments, growth rates of cells and plasmid copy numbers show positive correlations[1]. Thus, we could estimate the expression limit of a target protein by measuring the growth rate of cells containing the gTOW plasmid and the plasmid copy number within the cell under –Leu–Ura conditions. The growth rate and copy number under –Ura conditions also reflects the expression limits of the target protein[1].

Supplementary Tables

Table S1 (an Excel file). Localization signals and modifications used in this study with amino acid sequences (related to Table 1)

Table S2 (an Excel file). Genes whose expressions were changed upon high-level expressions of modified GFPs (related to Figure 2)

Table S3 (an Excel file). Plasmids used in this study

Supplementary Figures

Figure S1

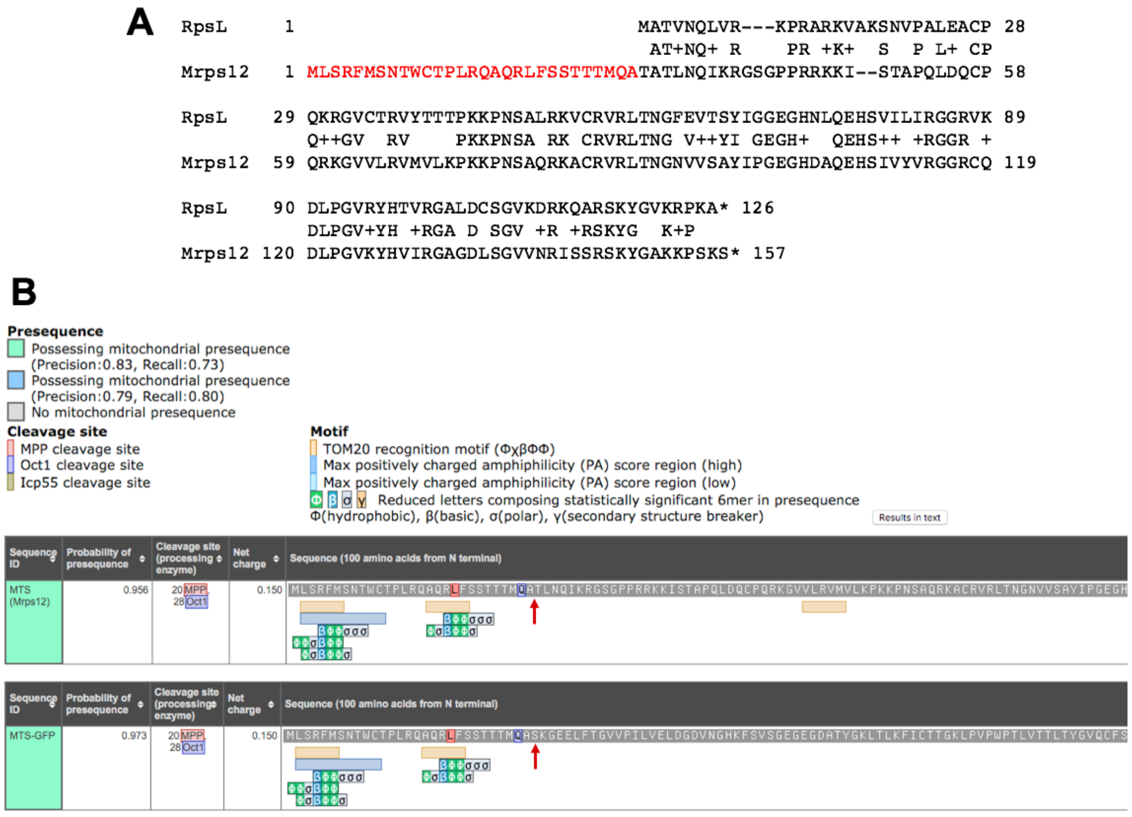

Figure S1. Characterization of MTS from Mrps12

A) Alignment of amino acid sequences between *E. coli* RpsL and *S. cerevisiae* Mrps12. RpsL and Mrps12 are considered to share the same ancestral bacterial ribosomal protein, and Mrps12 has evolved to be a mitochondrial protein[3]. We thus can consider that the extra N-terminal amino acid sequence as the mitochondrial targeting signal (MTS, indicated in red letters). RpsL sequence was obtained from EcoliWiki (http://ecoliwiki.net), and Mrps12 sequence was obtained from *Saccharomyces* Genome Database (http://www.yeastgenome.org). **B**) Probabilities of presequences are quite high in Mrps12 (0.956) and MTS-GFP (0.973). Predictions of MTSs were performed using MitoFates algorithm (http://mitf.cbrc.jp/MitoFates), and the screenshots of the results were shown. Red arrowheads indicate the sites used to connect the MTS to GFP.

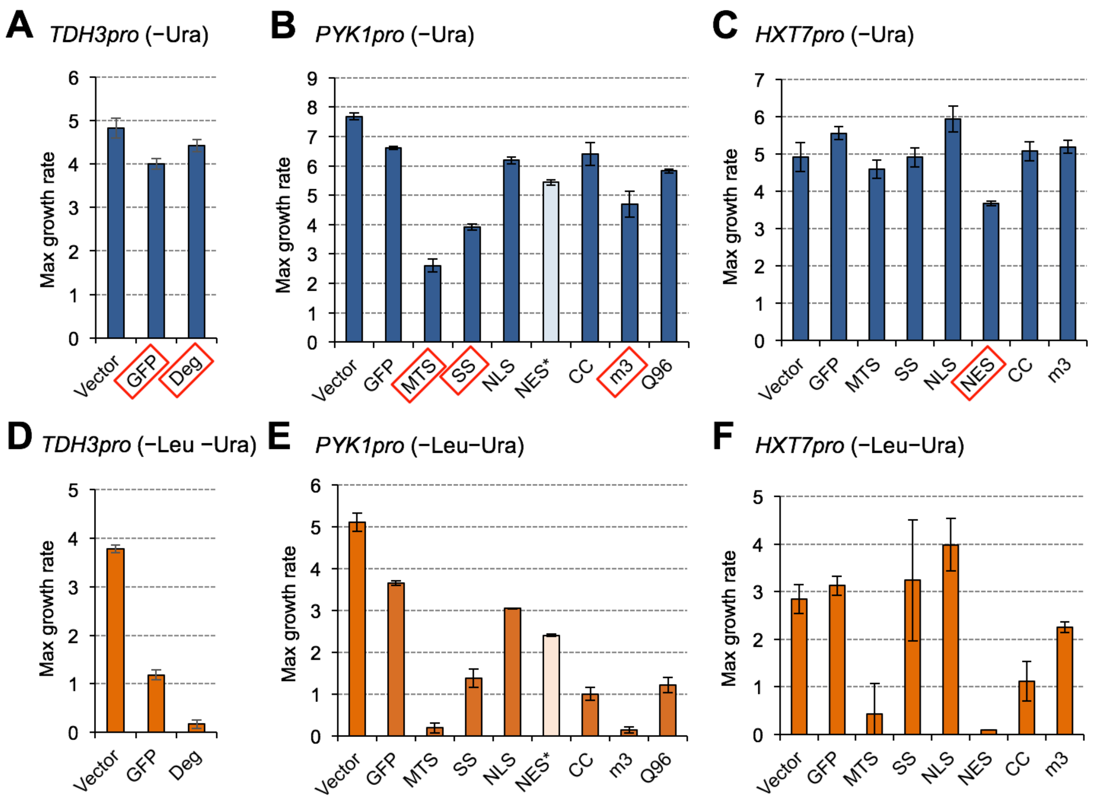

Figure S2. Growth rates of cells harboring gTOW plasmids containing modified GFPs (related to Figures 1 and 2)

Promoters and conditions are shown at the top of plots. Error bars represent standard deviations of independent measurements. Samples indicated with red squares were analyzed in the microarray analysis.

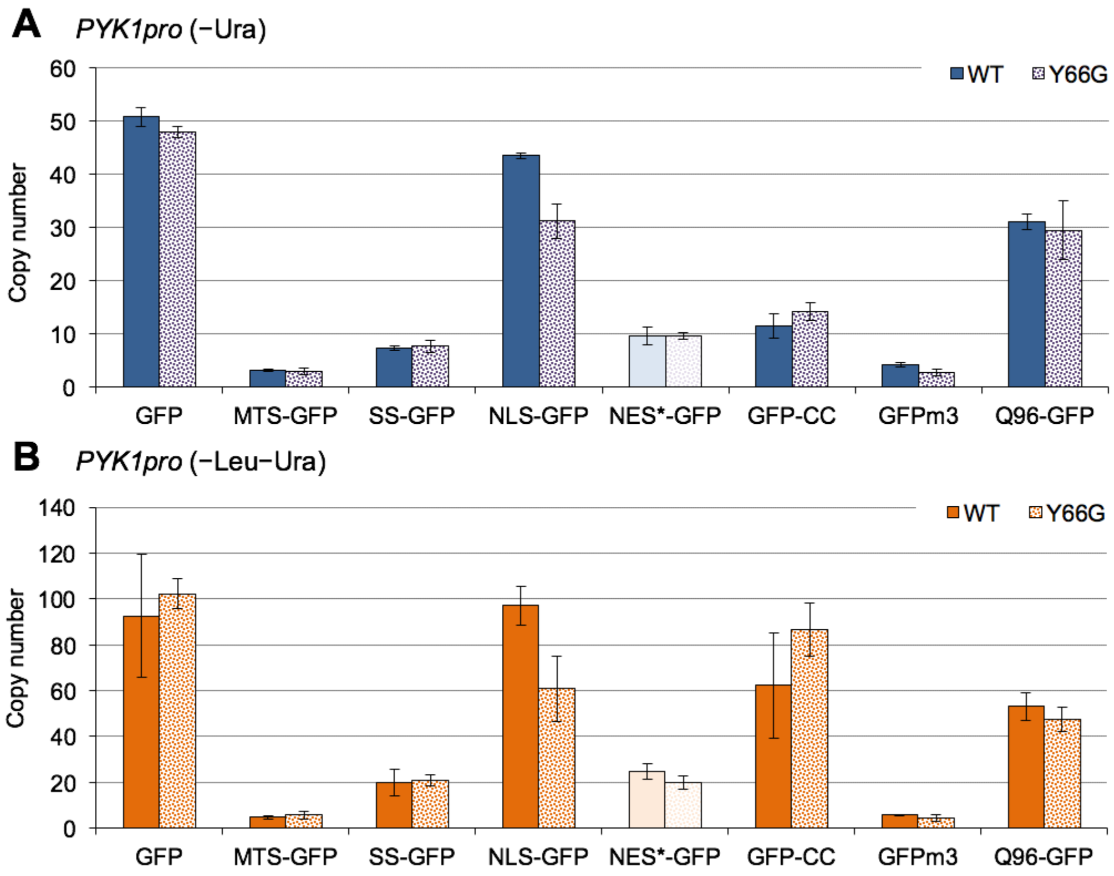

Figure S3. Copy-number limits of modified GFP with fluorophore mutations (Y66G)

Promoters and conditions are shown at the top of plots. Error bars represent standard deviations of independent measurements.

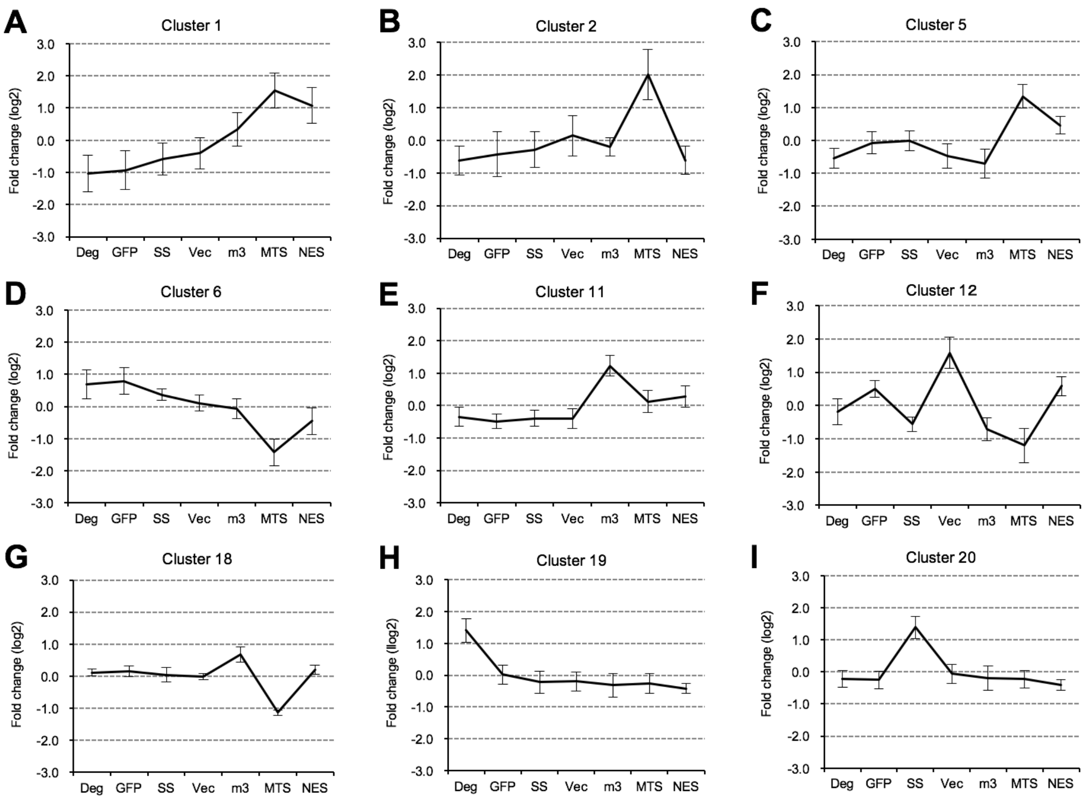

Figure S4. Expression patterns of characteristic clusters (related to Figure 2)

Averages and standard deviations of fold changes (log_2) of genes in the clusters.

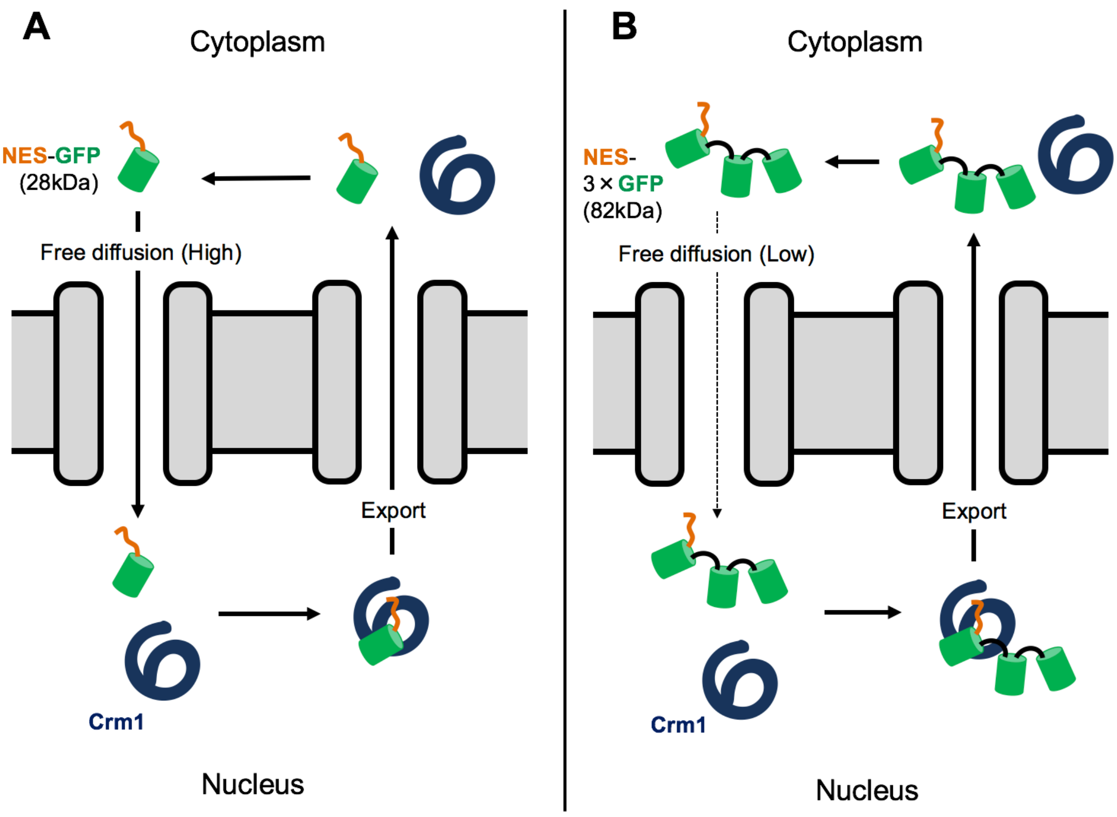

Figure S5. Working models explaining the difference in expression limits between monomeric and 3×GFP with NES (related to Figure 3)

A) The monomeric GFP with NES freely diffuses into the nucleus because it is larger than the size exclusion limit of the nuclear pore and is exported from the nucleus by Crm1. This free import–Crm1-dependent export cycle causes the overload of the Crm1-dependent nuclear export process. **B**) The 3×GFP with NES does not diffuse into the nucleus because it is larger than the size exclusion limit of the nuclear pore. When it is imported into the nucleus, it is exported by Crm1. Thus, the import–export cycle is slower than that of the monomeric GFP with NES and causes little overload of the nuclear export process.

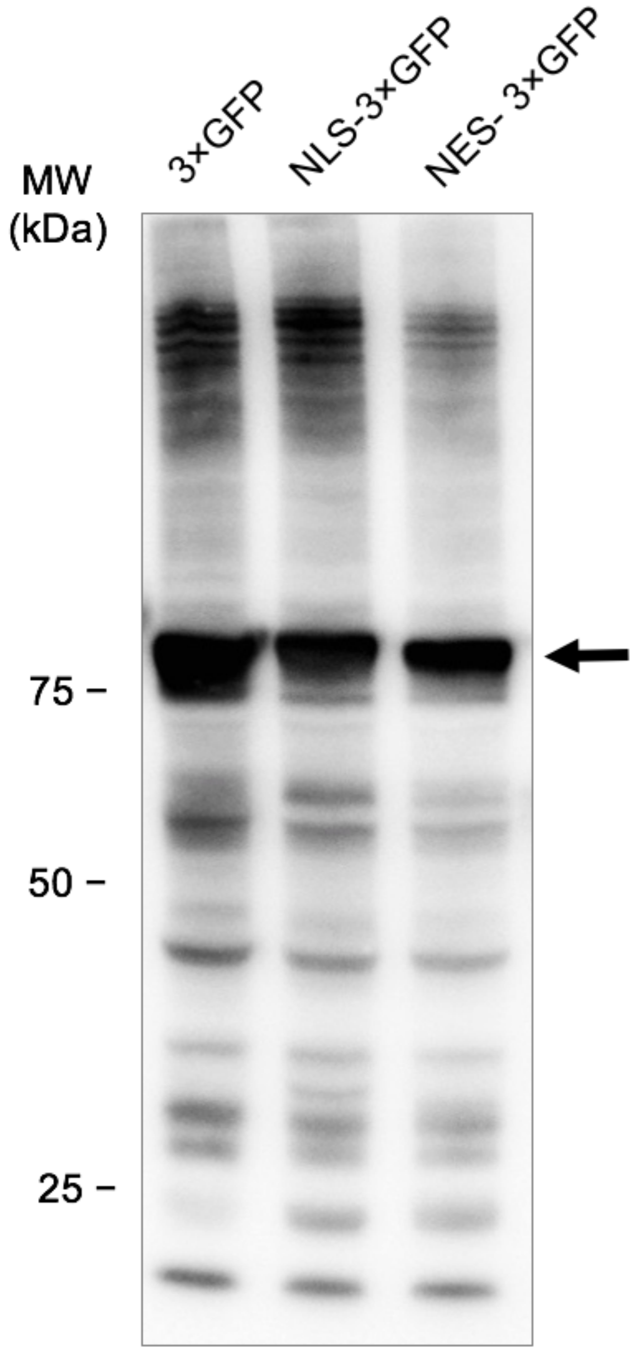

Figure S6. Western blots analysis of 3×GFP, NLS-3×GFP, and NES-3×GFP using anti-GFP antibodies

Protein samples were prepared from 32 μL of cells with OD_{600} 1.0 cultured in –Ura medium. Molecular weight (kDa) is shown on the left. The arrowhead indicates the expected molecular weight of 3×GFP (80.5 kDa).

Figure S7

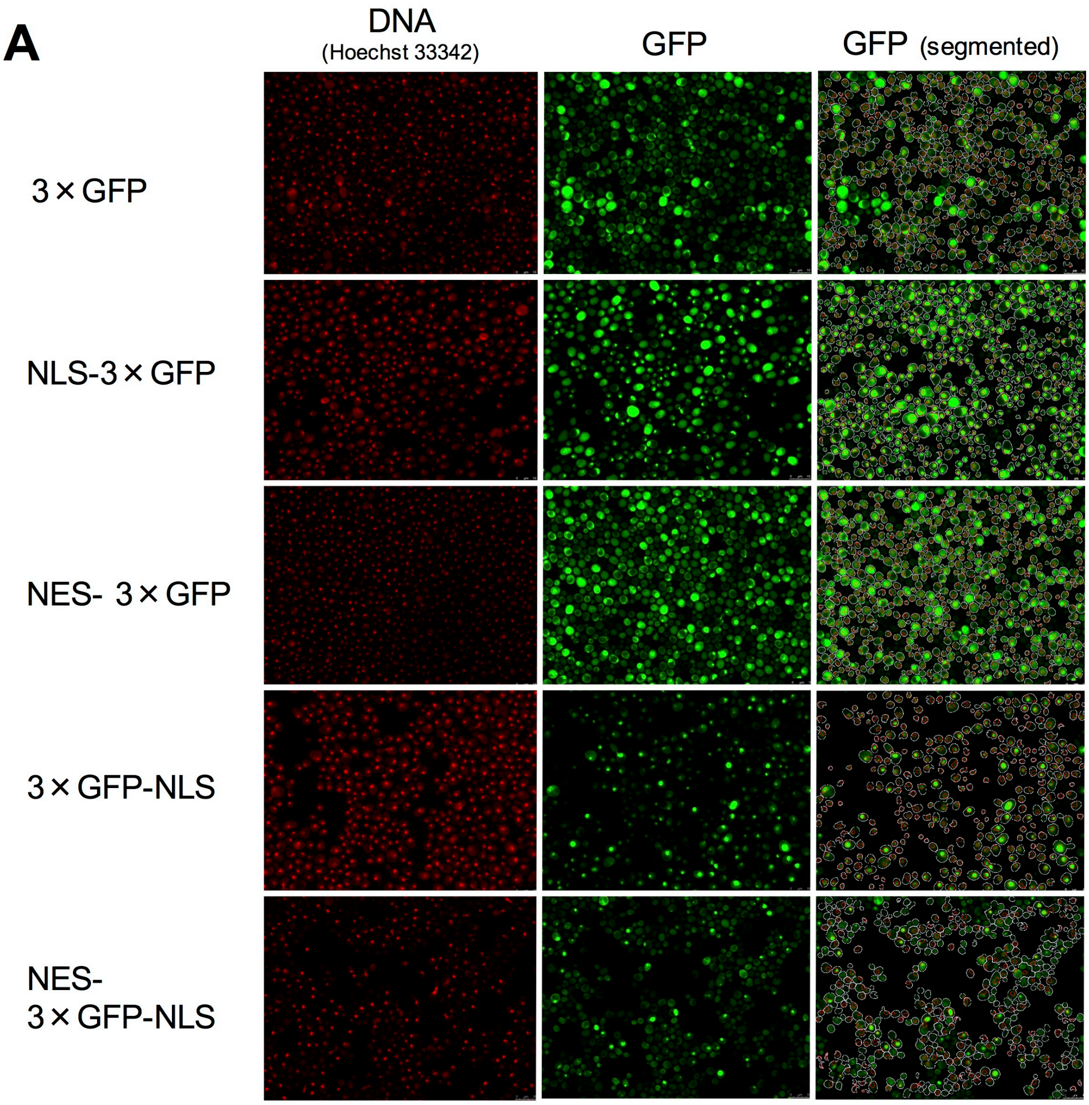
A
DNA
(Hoechst 33342)
GFP
GFP (segmented)
3×GFP
NLS-3×GFP
NES- 3×GFP
3×GFP-NLS
NES-
3×GFP-NLS

Figure S7 continues

B

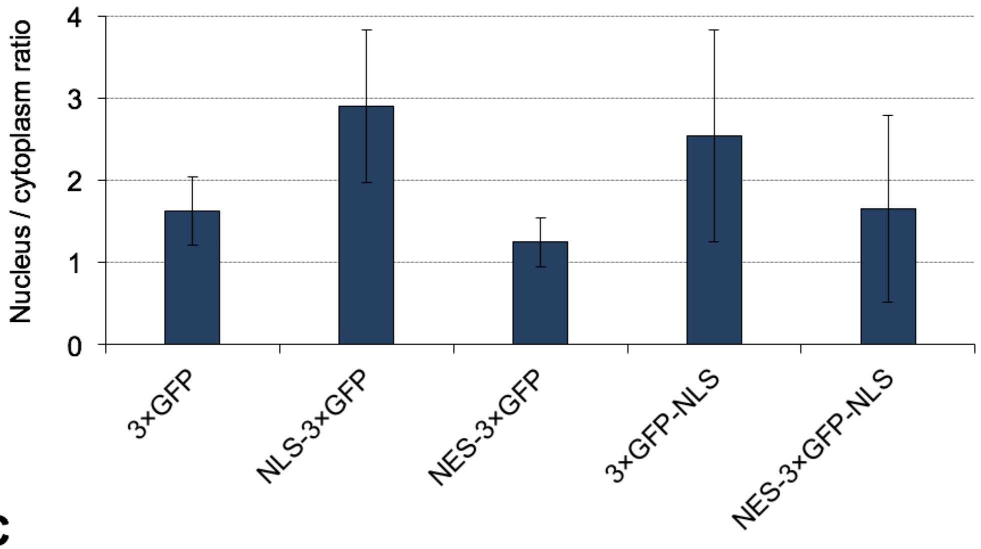

C

	NLS-3 × GFP	NES-3 × GFP	3 × GFP-NLS	NES-3 × GFP-NLS
3 × GFP	4.6E-96	1.1E-50	1.0E-35	6.6E-01
NLS-3 × GFP		3.6E-179	9.0E-05	3.6E-35
NES-3 × GFP			2.3E-82	3.0E-13
3 × GFP-NLS				7.3E-14

Figure S7. Evaluation of NLS and NES functionalities attached to 3×GFP

The functionalities of NLS and NES were confirmed by quantification of nucleus/cytoplasm ratios of GFP intensities. NES functionality was also confirmed by the counteracting activity against C-terminal NLS (compare 3×GFP-NLS and NES-3×GFP-NLS. **A**) Segmentation of nuclear and cytoplasmic regions. Nuclear regions were segmented using DNA images, and cellular regions were segmented using GFP images. Cytoplasmic regions were segmented by subtracting the nuclear region from the cytoplasmic region of each cell. **B**) Nucleus/cytoplasm ratios of 3×GFPs with localization signals. For each construct, the mean and standard deviation (shown as the error bar) of pixel intensity values in the nuclear and cytoplasmic regions were calculated from >200 cells. **C**) *p*-values of Student's *t* test between different constructs. Measurements were performed using the CellProfiler software[4]. Analytical pipeline (.cppipe file) used in this study is provided upon request.

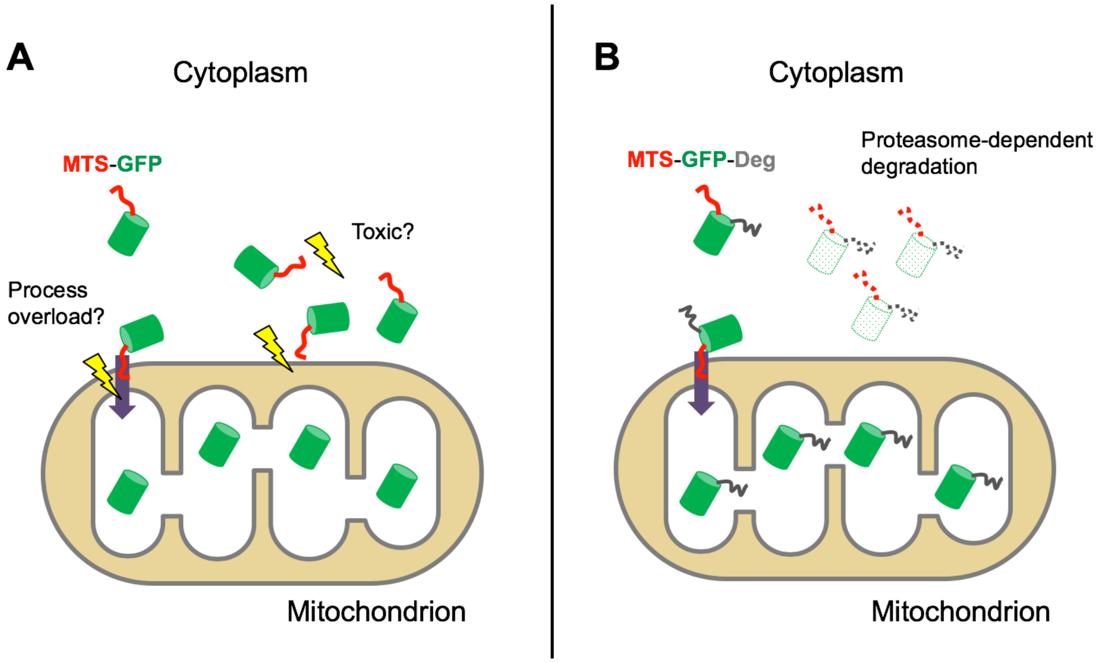

Figure S8. A working model explaining the determinants of expression limit of MTS-GFP (related to Figure 5)

A) MTS-GFP overloads mitochondrial transport machinery or the precursor form causes cellular toxicity, probably owing to the properties of MTS amino acid sequence. **B**) By attachment of the proteasome-dependent degron (Deg), the cytoplasmic precursor form of MTS-GFP is selectively digested.

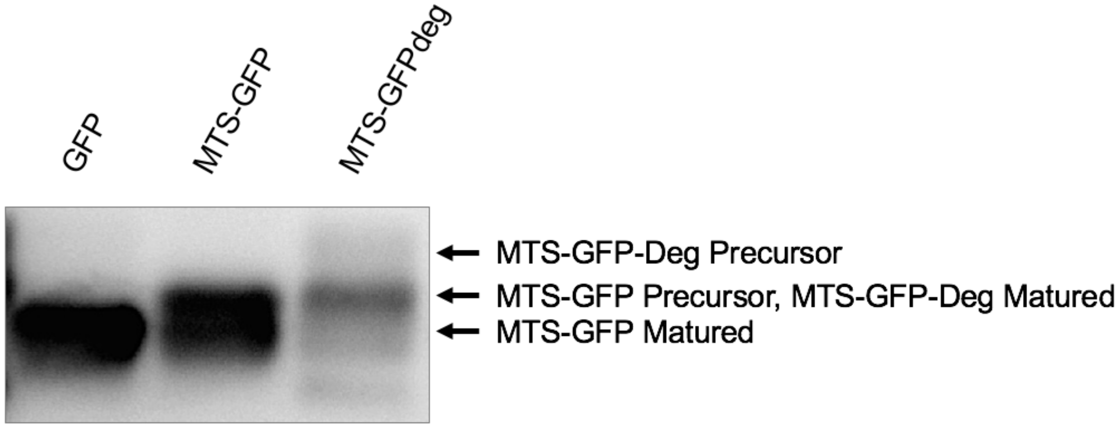

Figure S9. Western blots analysis of GFP, MTS-GFP, and MTS-GFP-Deg using anti-GFP antibodies

Protein samples were prepared cells cultured in –Ura medium. Protein sample from 5 μL of cells with OD_{600} 1.0 were loaded in the GFP experiment. Protein sample from 50 μL of cells with OD_{600} 1.0 were loaded in the MTS-GFP and MTS-GFP-Deg experiments. The arrowheads indicate the predicted precursor and matured forms.

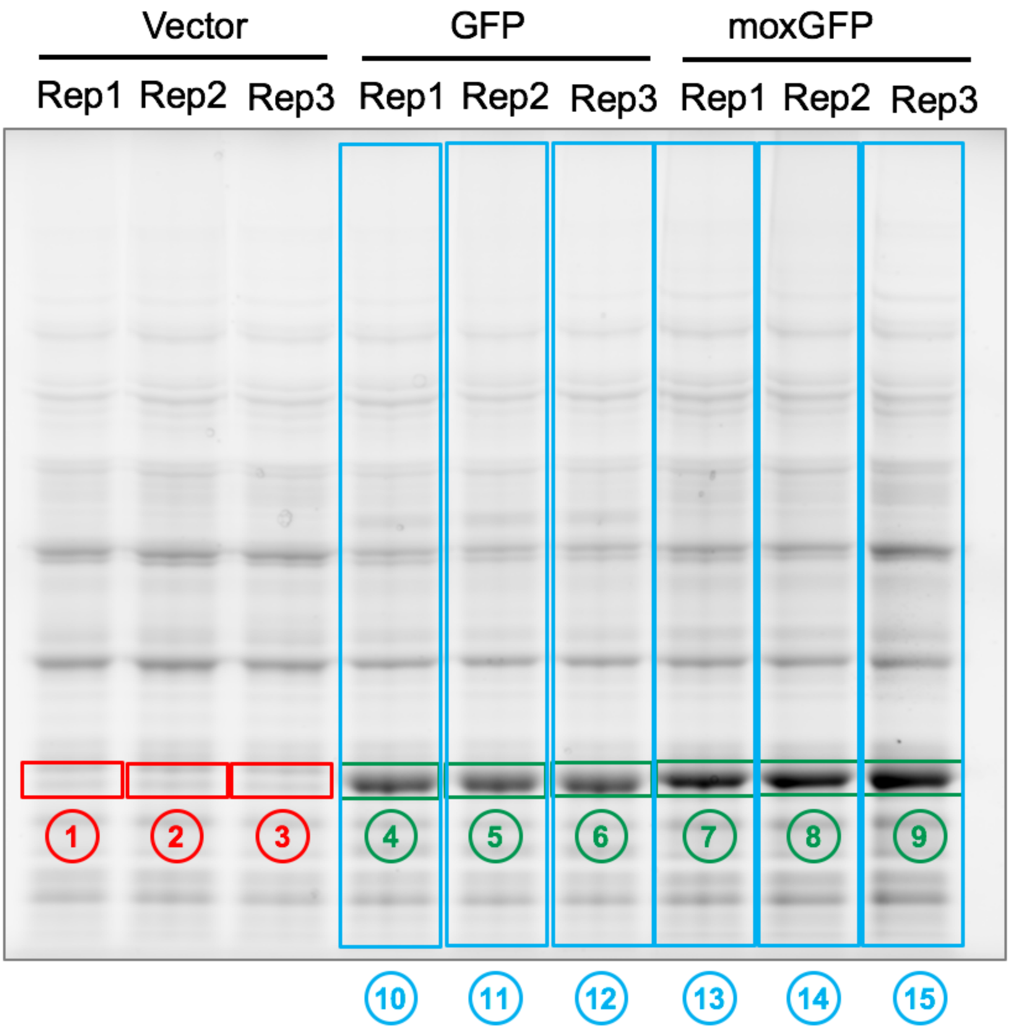

Figure S10. Measurement of GFP levels relative to the total proteins (related to Figure 6)

SDS-PAGE separated proteins pre-labeled with a fluorescent dye (Ezlabel FluoroNeo) that labels primary and secondary amines, and areas measured are shown. Volumes of squared areas were measured as the sums of the pixel values in the areas after subtraction of the background. The measurement was performed as follows: 1) Background in GFP bands (**B**) was calculated as the average volume of ①, ②, and ③; 2) Percentage volume of each GFP band with respect to total protein was calculated. For example, % total protein of GFP Rep1 was calculated as (④–**B**)/(⑩–④+**B**) * 100, and; 3) The mean and standard deviation of a biological triplicate experiment were calculated.

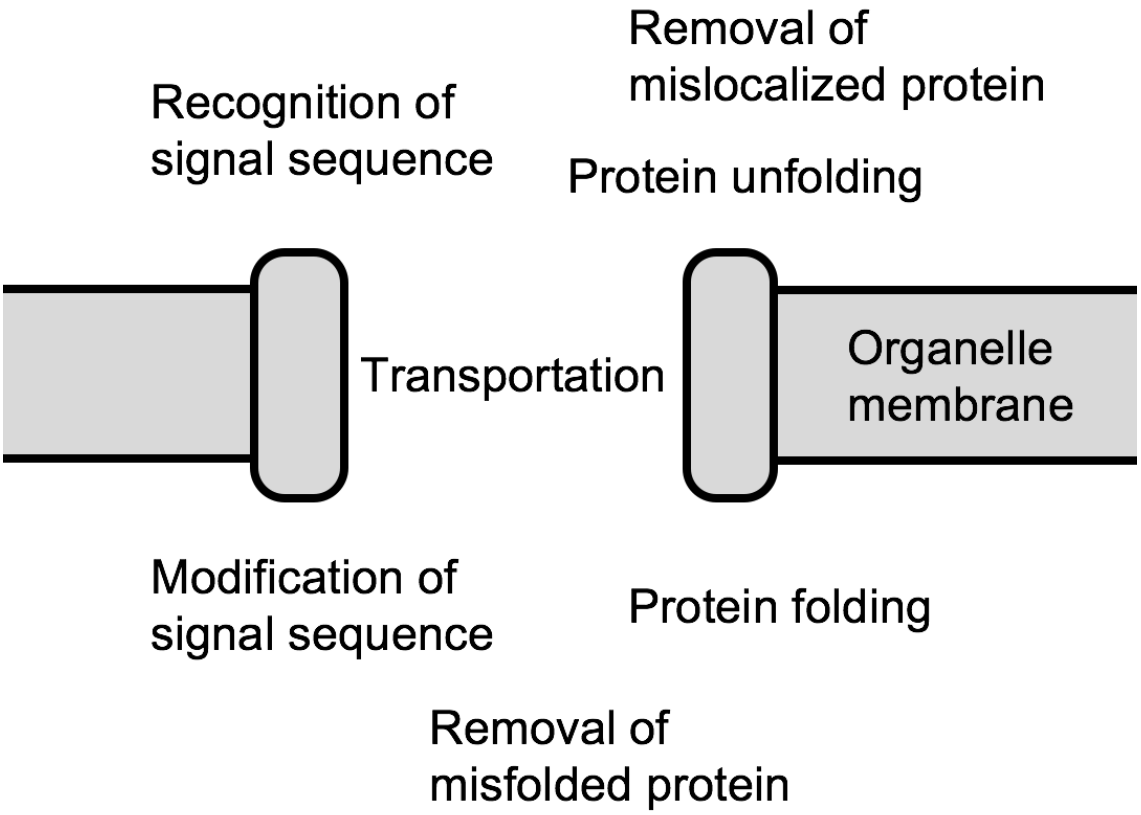

Figure S11. Dissection of localization processes

Localization processes can be dissected into individual resource-consuming processes.

Original blot image used in Figure 5C

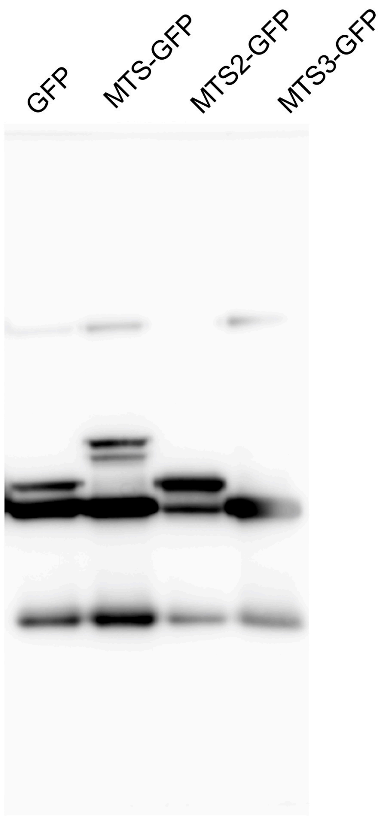

Original blotimages used in Figure 6C

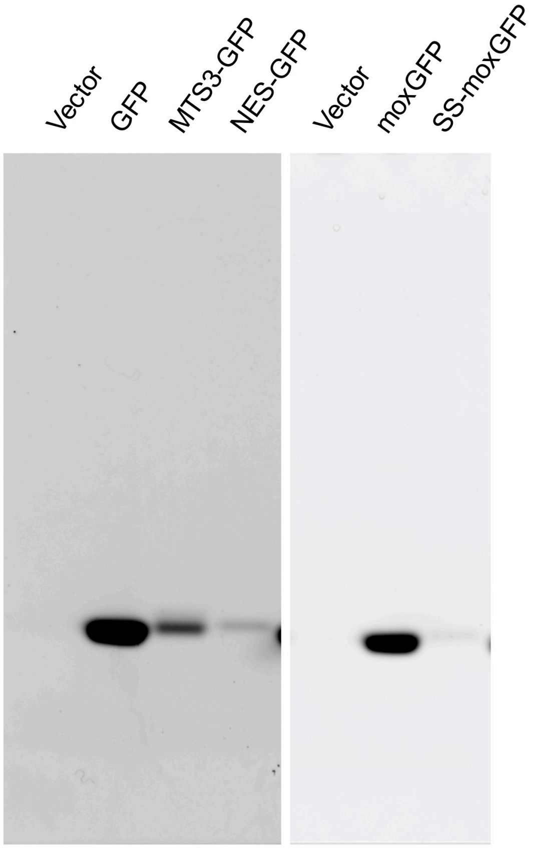

Figure S12. Original blot images used in Figure 5C and Figure 6C

Supplementary References

1 Moriya, H., Shimizu-Yoshida, Y. & Kitano, H. In vivo robustness analysis of cell division cycle genes in Saccharomyces cerevisiae. *Plos Genetics* **2**, 1034-1045, doi:10.1371/journal.pgen.0020111 (2006).

2 Moriya, H., Makanae, K., Watanabe, K., Chino, A. & Shimizu-Yoshida, Y. Robustness analysis of cellular systems using the genetic tug-of-war method. *Molecular Biosystems* **8**, 2513-2522, doi:10.1039/c2mb25100k (2012).

3 Fujita, K., Horie, T. & Isono, K. Cross-genomic analysis of the translational systems of various organisms. *J Ind Microbiol Biotechnol* **27**, 163-169 (2001).

4 Carpenter, A. E. *et al.* CellProfiler: image analysis software for identifying and quantifying cell phenotypes. *Genome Biol* **7**, R100, doi:10.1186/gb-2006-7-10-r100 (2006).
